# Temperature and Inoculum Origin Influence the Performance of Ex-Situ Biological Hydrogen Methanation

**DOI:** 10.3390/molecules25235665

**Published:** 2020-12-01

**Authors:** Noémie Figeac, Eric Trably, Nicolas Bernet, Jean-Philippe Delgenès, Renaud Escudié

**Affiliations:** 1 INRAE, University of Montpellier, LBE, 102 Avenue des Etangs, 11100 Narbonne, France; noemie.figeac@inrae.fr (N.F.); eric.trably@inrae.fr (E.T.); nicolas.bernet@inrae.fr (N.B.); jean-philippe.delgenes@inrae.fr (J.-P.D.); 2 French Environment and Energy Management Agency, 20 Avenue du Grésillé- BP 90406, CEDEX 01, 49004 Angers, France

**Keywords:** biogas upgrading, power-to-gas, hydrogenotrophic methanogens, mixed culture

## Abstract

The conversion of H_2_ into methane can be carried out by microorganisms in a process so-called biomethanation. In ex-situ biomethanation H_2_ and CO_2_ gas are exogenous to the system. One of the main limitations of the biomethanation process is the low gas-liquid transfer rate and solubility of H_2_ which are strongly influenced by the temperature. Hydrogenotrophic methanogens that are responsible for the biomethanation reaction are also very sensitive to temperature variations. The aim of this work was to evaluate the impact of temperature on batch biomethanation process in mixed culture. The performances of mesophilic and thermophilic inocula were assessed at 4 temperatures (24, 35, 55 and 65 °C). A negative impact of the low temperature (24 °C) was observed on microbial kinetics. Although methane production rate was higher at 55 and 65 °C (respectively 290 ± 55 and 309 ± 109 mL CH_4_/L.day for the mesophilic inoculum) than at 24 and 35 °C (respectively 156 ± 41 and 253 ± 51 mL CH_4_/L.day), the instability of the system substantially increased, likely because of a strong dominance of only *Methanothermobacter* species. Considering the maximal methane production rates and their stability all along the experiments, an optimal temperature range of 35 °C or 55 °C is recommended to operate ex-situ biomethanation process.

## 1. Introduction

The European Commission’s Renewable Energy Road Map 2050 estimates that 55% of the total energy consumed in 2050 will be produced from renewable energy sources within the European Union (EU) [1]. Wind and solar energy will represent an important part of the renewable energy in EU. As an illustration, the electricity generation from wind farms in EU (28 members) increased from 377 TWh in 2018 to 425 TWh in 2019 [2]. Since energy production from wind and solar farms is intermittent, electricity storage before injection to the power grid will be required in the future to ensure a good balance between electricity production and consumption [3]. In the power-to-gas (PtG) concept, the electricity surplus can be converted into hydrogen (H_2_) by water electrolysis. However, H_2_ has a low volumetric energy density and its transportation and storage are still technological issues [4]. As an alternative, H_2_ can be further combined with carbon dioxide (CO_2_) to produce methane (CH_4_) through a methanation reaction [5].

Two technologies can be used to perform the methanation reaction. The so-called “catalytic” methanation, based on the Sabatier reaction, is carried out using chemical catalysts such as nickel at high temperatures (250–450 °C) [6,7]. The biological methanation (also called biomethanation) is based on microorganisms, the conversion being operated at moderate temperatures and pressures (35–65 °C and atmospheric pressure up to 10 bars, respectively) [8,9,10]. In addition, when performed on biogas issued from anaerobic digestion (AD) plants, biological methanation can be considered as a substitute of a CO_2_ purifier through biogas upgrading [7,11].

Mechanistically, the biological methanation reaction corresponds to one of the last biological reactions of the anaerobic digestion process, as methanogenic *archaea* are the sole microorganisms able to convert hydrogen (H_2_) and carbon dioxide (CO_2_) into CH_4_ [12]. The main advantage of the biological methanation process is the low energy cost if compared to catalytic methanation [7,13]. Since the CO_2_ content in the AD biogas ranges usually between 25 and 48% [5], biological methanation is an adequate technology for biogas upgrading into biomethane (up to 98% of CH_4_) [14]. In addition to the storage and buffering capacity in a PtG concept, this technology makes the biogas enriched in CH_4_ and susceptible to be injected into the existing gas distribution grid or use as fuel in the transportation sector [13]. 

The biological methanation reaction in mixed cultures can be carried out through two microbial pathways: (i) a direct pathway corresponding to the conversion of H_2_ and CO_2_ into CH_4_ by hydrogenotrophic methanogens and (ii) an indirect pathway where H_2_ and CO_2_ are first converted into acetate by homoacetogenic bacteria and then acetate is converted into CH_4_ and CO_2_ by acetotrophic methanogens [11,13,15]. Several studies have previously investigated the effects on the biological methanation process of the injection of hydrogen and exogenous CO_2_ into an anaerobic reactor. The present study investigated ex-situ biomethanation since volumetric methane production rates are higher than for in-situ mode [16] and since fewer reactions are involve, maintain the parameters stable is easier [17]. One of the most important limitations relies on the gas-liquid transfer of H_2_ [18,19,20]. The gas-liquid mass transfer rate Rt can be descripted by the following Equation (1):

Rt = kLa × (CL* − CL)(1)

where kLa is the transfer volume coefficient, CL* is the saturation concentration and CL is the dissolved concentration. One way to increase the H_2_ gas–liquid mass transfer rate is to modify the gas transfer coefficient (k_L_a). k_L_a strongly depends on the reactor configuration and can be increased by modifying the mixing speed [19], gas flow [21] or the gas sparger device [22,23]. Another way is to increase the gas solubility and thus the driving force (i.e., CL* − CL) of gas-liquid mass transfer rate, by modifying pressure and temperature. [24]. Indeed, even if the temperature can modify the kLa through the modification of soluble gas diffusion coefficient and the viscosity of the medium, lowering the temperature of the reactor can increase drastically the solubility of H_2_. However, temperature affects also the carbon to CH_4_ bioconversion pathway [7]. Under thermophilic conditions, hydrogenotrophic methanogens are more active [25], while homoacetogens are better adapted to lower temperatures [26]. The relative contribution of hydrogenotrophic methanogenesis and the absolute rate of acetoclastic methanogenesis decreases with temperature [26]. For in-situ biomethanation, Zhu et al. reported that indirect pathway via acetotrophs was most used under mesophilic conditions, while the direct hydrogenotrophic methanogenesis pathway predominated under thermophilic condition [27]. Previous works showed an optimal growth of hydrogenotrophic methanogens at temperatures between 55 °C and 70 °C [28,29,30]. Other previous works on ex-situ biomethanation demonstrated that the operating temperature was important for the biomethanation performances [31]. Luo and Angelidaki showed that a process operated at 55 °C with an inoculum sampled from a thermophilic anaerobic digester was twice as fast as a process operated at 37 °C with an inoculum sampled from a mesophilic digester [19]. Consistently, Guneratnam et al. demonstrated that a biological methanation system exhibited higher methane content and productivity at 65 °C than 55 °C with an inoculum sampled from an anaerobic digester operated at 55 °C during the 12 first hours [29]. Dong et al. reported also that the methanogenic activity increased with increasing temperature by carrying out experiments at 55 °C, 65 °C and 70 °C [32].

In addition, the temperature can also impact the microbial community structure on ex-situ biomethanation. Previous studies based on H_2_-assisted biogas upgrading showed that H_2_ addition could affect the microbial community composition by increasing both the abundance in hydrogenotrophic methanogens and the bacterial syntrophic interactions with methanogenic *archaea* [10,18,29,33]. Bassani et al. proposed a two-stage system composed of a first anaerobic reactor followed by an ex-situ biological methanation reactor [18]. These authors observed a microbial shift toward the hydrogenotrophic pathway with a significant increase in abundance (around 3 folds) of hydrogenotrophic methanogens, and a sharp decrease in acetoclastic methanogens. Guneratnam et al.) also investigated the archaeal community structure in a biological methanation process and showed that *Methanothermobacter* species dominated the microbial communities in thermophilic biological methanation systems. Interestingly, *Methanothermobacter* sp. was also present in high proportion in the inoculum originated from a thermophilic digester [29]. Chen et al. compared thermophilic and extreme-thermophilic conditions. They showed that hydrogenotrophic methanogens abundance increased with increasing temperature and *Methanothermobacter* sp. was even more enriched at higher temperature (70 °C) than at 55 °C [34]. Moreover, Braga Nan et al. compared seven inocula and demonstrated that the composition of the inocula influenced the methane production [8]. Only this study compared different inocula at the same temperature and thus can determine if methane production is linked to microbial composition. However, no study has tested a single inoculum over a large and wide range of temperature. In addition, test two inocula would make it possible to determine if the higher methane production of inocula at high temperatures were due to the microbial composition or the incubation temperature of the reactors.

Because of the antagonist effects of the temperature on H_2_ gas–liquid mass transfer rates and on microbial kinetics, the objective of this study was to assess the influence of temperature on the performances of biological methanation process in mixed culture and evaluate the role and the impact of the initial microbial communities. A range of temperatures from 24 °C to 65 °C was applied in a semi-continuous mode. Two different inocula, mesophilic and thermophilic, were tested both at high (55 and 65 °C) and low temperatures (24 and 35 °C). The performances were evaluated considering the maximal methane production rates and their stability all along the experiments. The microbial community compositions at the start and at the end of the experiments were analyzed to characterize the microbial community changes driven by the biological methanation conditions.

## 2. Results and Discussion

### 2.1. Effect of Temperature and Inoculum Origin on Reactor Performances

Since the experiments were performed in two different runs (run 1 for temperatures at 35 and 55 °C and run 2 for temperatures at 24 and 65 °C), the two mesophilic and thermophilic microbial inocula were analyzed at the beginning of each run. The microbial community composition was very similar for the two samples, whatever the inoculum (mesophilic or thermophilic), although the amount of the *archaea* was slightly higher in the second sample (data in Supplementary Materials).

Both thermophilic and mesophilic inocula were not pre-cultured before H_2_ injection to evaluate the adaptation phase of the indigenous microbial community. For both inocula (mesophilic and thermophilic) and at all temperatures (24, 35, 55 and 65 °C), CH_4_ production and H_2_ consumption started just after the first H_2_ injection, as consistently observed in the literature [8,17,28]. Such immediate H_2_ consumption indicated that the H_2_ concentration was low enough to not cause inhibition of the activity of the hydrogenotrophic methanogens.

#### 2.1.1. Maximal CH_4_ Production Rates

**Figure 1:**
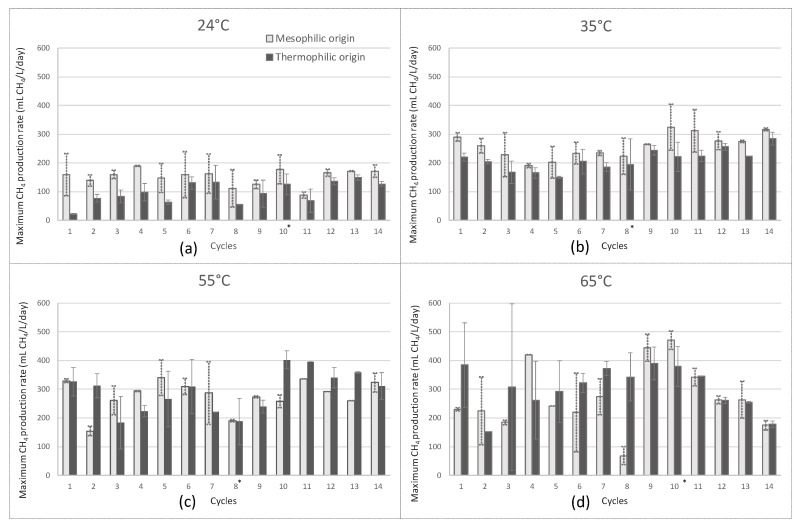
Maximum CH_4_ production rate (mLCH_4_/L.d) during 14 cycles at 24 °C (left top (**a**)), at 35 °C (right top (**b**)), at 55 °C (left bottom (**c**)) and at 65 °C (right bottom (**d**)) for the ex-situ biological methanation reactors. The black bars represent the reactors inoculated with a thermophilic inoculum and the white bars correspond to the reactors were inoculated with a mesophilic inoculum. * These bars are not representative due to the change of septum.

For each cycle, the maximal CH_4_ production rate was estimated from the dynamics of reactor pressure and gas composition. In Figure 1, the maximal CH_4_ production rates are presented for each cycle and for all operating conditions. The maximal rates were 189, 324, 402 and 471 mL/L.day at 24, 35, 55 and 65 °C, respectively. Overall, the increasing temperature had a positive effect on the CH_4_ production rate. 

**Figure 2:**
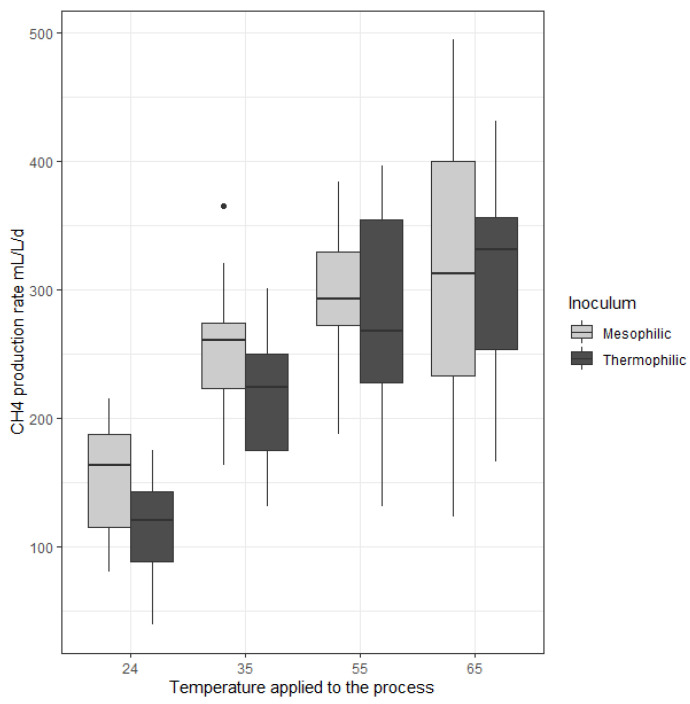
Influence of temperature and inoculum source of the maximal CH_4_ production rate. The horizontal black lines represent the median and the vertical ones represent the dispersion of the values. The white boxes represent the reactors seeded with a mesophilic inoculum and the black boxes the reactors seeded with a thermophilic inoculum.

The impact of temperature and inoculum origin on the maximum methane production rate are shown in Figure 2. Overall, the increasing temperature had a positive effect on the maximal CH_4_ production rate whatever the type of inoculum. As an example, for the mesophilic inoculum, average values of 156 ± 41 mL/L.day mL/L.day and 309 ± 109 mL/L.day mL/L.day were observed for the temperatures 24 and 65 °C, respectively (Table 1). The robustness of the process is represented by the dispersion magnitude of the data. Interestingly, the dispersion of the CH_4_ production rate increased with the increasing temperatures. As an illustration, for the mesophilic inoculum the standard deviation at 65 °C was twice higher than at 55 °C, i.e., 55 and 109 mL/L.day, respectively. Therefore, it was concluded that CH_4_ production was less stable at high temperatures, whatever the inoculum.

In order to compare the maximum CH_4_ production rates from the different operating conditions statistical analyses were carried out. A Kruskal Wallis statistical test (*p*-value < 0.05) was performed to evaluate whether at least one temperature assay had a different methane production rate than the others. However, this test does not allow to determine which assay is different to the others. For each temperature, a Dunn test, i.e., a pairwise multiple-comparison test, was thereafter performed to investigate the difference between the temperatures two by two. As a result, for the thermophilic inoculum-based data set, 24 °C was different from the other temperatures with a *p* value < 0.05. With the same maximum *p* value, 35 and 65 °C assays were different. The analysis between 35 and 55 °C had a *p* value of 0.06 which was very close to be significant and the values at 55 and 65 °C were similar with a *p* value of 0.58 which was strongly not significant. For the mesophilic inoculum-based data set, only 24 °C was different from the other temperatures (*p*-value < 0.05), and no statistical difference was observed between the other temperatures. In conclusion, the maximal CH_4_ production rates under mesophilic/thermophilic temperatures were always statistically higher than at 24 °C. The higher instability at high temperatures, i.e., the higher variability of the values, could explain why the *p* values were not significant. 

Since two different inocula are considered and two groups had to be compared, Student tests were performed to analyze the effect of the microbial inoculum. For each temperature, the maximal CH_4_ production rates of the two inocula were compared. The results showed that the inoculum origin had only an impact at 24 °C and 35 °C with a *p* value < 0.05, the mesophilic inoculum being associated to a higher maximal CH_4_ production rate. For 55 °C and 65 °C, the inoculum origin showed no influence on methane production rates (*p*-value > 0.7).

Luo and Angelidaki compared the biological methanation performances of an inoculum sampled from a mesophilic anaerobic digester operated at 35 °C with another inoculum issued from a thermophilic anaerobic digester operated at 55 °C [19]. They observed a two-fold increase of the maximal CH_4_ production rate from 35 °C to 55 °C (from 1900 mL/L.day to 3800 mL/L.day, respectively) when the inoculum adapted to the working temperature. Similarly, Bassani et al. reported that the CH_4_ production rate increased from 100 mL/L.day to 359 mL/L.day between 35 °C and 55 °C, respectively [18]. Meanwhile, Guneratnam et al. used the same inoculum (from a thermophilic reactor at 55 °C) at two different temperatures (55 °C and 65 °C) [29]. During the first 12h after gas feeding, the performances at 65 °C were better than at 55 °C, with an increase of the maximal methane production rate from 200 to 560 mL CH_4_/L.day. However, within a 24 h period, the maximal methane production rates became closely similar (450 and 400 mL CH_4_/L.day at 55 °C and at 65 °C, respectively). Similarly, Dong et al. observed no significant difference in the methane production between reactors inoculated with the same inoculum at three temperatures: 55, 65 and 70 °C [32]. Braga Nan et al. observed different methane production depending of inoculum origin [8]. These previous works confirmed that the maximal CH_4_ production rates are globally higher at high temperatures (i.e., 55 and 65 °C) with no significant differences between 55 and 65 °C. Nonetheless, as shown in the present study, the maximal methane production rates are also strongly dependent on the inoculum origin at low temperatures. Thus, the conclusions of the present work are consistent with these studies and indicate that an inoculum with a mesophilic origin can present a better methane production than an inoculum with a thermophilic origin at low temperatures but, interestingly, with no difference at high temperatures.

Even though gas transfer was likely enhanced at 24 °C compared to higher temperatures, the microbial activity was however significantly lowered. Therefore, the negative effect of the low temperature on the microbial kinetics had a higher impact in biological methanation than on H_2_ gas–liquid mass transfer rates. In counterpart, the instability of the system at high temperatures hampered the effect of the microbial inoculum with a high variability of the values. 

**Figure 3:**
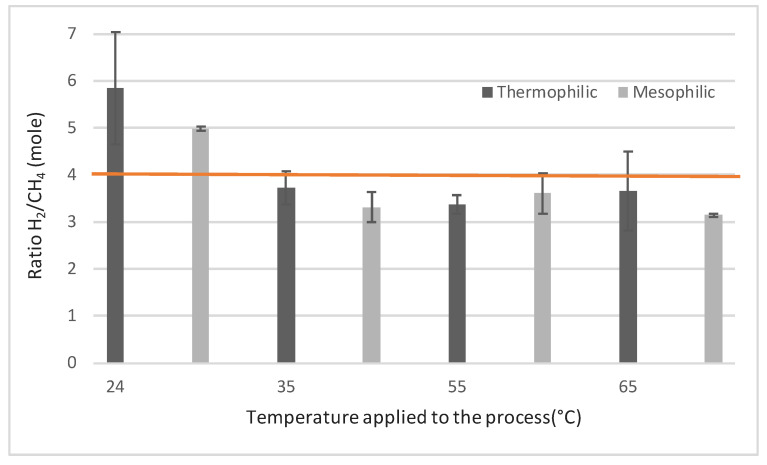
Evolution of the ratio H_2_ consumed mole per CH_4_ produced mole with temperature. The horizontal orange line represents the stoichiometric value (4 moles of H_2_ consumed per mole of CH_4_ produced).

From a process point of view, the conversion yields of the substrate (i.e., hydrogen) into methane was also calculated. The H_2_/CH_4_ conversion yield expressed as the amount of H_2_ consumed per CH_4_ produced (moleH_2_/moleCH_4_) during the 14 cycles are shown in Figure 3. According to the stoichiometry of hydrogenotrophic methanogenesis, H_2_/CH_4_ yield should be equal to 4 without considering the cellular growth. H_2_/CH_4_ yields ranged between 3.1 and 5.8 (Figure 3). In particular, H_2_/CH_4_ yields were lower than 4 for the reactors operated at 35, 55 and 65 °C. One hypothesis for such overestimated methane production could be an excess in the CH_4_ production due to the degradation of residual organic matter from the inoculum, even though the endogenous CH_4_ production was evaluated in the blank reactors. Consistently, Luo and Angelidaki showed a H_2_/CH_4_ yield of about 3.5 over a 43-day period, and they attributed this low yield to an excess CH_4_ production from the residual organic matter contained in the inoculum [19]. For the reactors at 24 °C, and whatever the inoculum, the yield was always higher than 4, showing that hydrogen was not totally converted to methane. Hydrogen could have been used for other microbial reactions such as cell maintenance and growth, and the hydrogenotrophs could have been limited and needed an acclimation at this low temperature. During the first 20 days of operation, Rachbauer et al. also observed yields even higher than 7, but reached the stoichiometric value after 100 days [35]. They explained the high value of the initial yield by the acclimation of the microbial community to very low temperature. Another hypothesis is that hydrogen could have been used toward homoacetogenesis and in this case an accumulation of acetate would have been observed. These results were consistent with the COD mass balance estimation considering a reasonable measure variability error of 10%. At 24 °C, the methane missing could be attributed to the production of biomass and at the other temperatures the overestimated carbon conversion could be attributed to an endogenous methane production [8].

#### 2.1.2. VFA Accumulation

In AD, the microbial steps occurring prior to methanogenesis produce volatile fatty acids (VFA) by fermentation. In biological methanation, where only CO_2_ and H_2_ are used, VFA can only be produced by homoacetogenesis (transformation of CO_2_ and H_2_ into acetate). The final VFA concentrations, ranging between 0.34 and 1.64 g/L, and the final acetate concentration, ranging between 0.164 and 1.14 g/L, are shown in Table 1. The concentration of VFA (and acetate) was below the inhibition threshold since the acetate accumulation did not affect the methane production rate. Bassani et al. observed methane production for ex-situ biological methanation process containing up to 1.77 g/L of VFA [36]. Rachbauer et al. showed an effect on methane production at total acetate concentrations above 1.18 ± 0.15 g/L [37]. Neither temperature nor inoculum origin strongly affected the VFA production as shown by VFA accumulation during the stable period in Table 1. However, at 24 °C the percentage of produced VFA in regards to the produced metabolites (methane + VFA) was higher than for all other temperatures (ratio of about 14% compared to 2% for the other temperatures). Therefore, at 24 °C the metabolism was more directed towards the production of VFA than at other temperatures. At 35, 55 and 65 °C, the reactors produced almost only methane (maximum 2% in average of the total produced metabolites was VFA) as observed by Braga Nan et al. for when inoculating bioreactor with leachate from a cattle manure dry anaerobic digester [8]. However, they obtained 81% and 78% of methane in average from the total produced metabolites, respectively, from the reactors inoculated with a granular sludge coming from an up-flow anaerobic sludge blanket (UASB) reactor treating paper mill waste (inoculum GS) and from the reactors inoculated with a sludge from an anaerobic digester treating aerobic sludge.

VFA accumulation occurred during the first 14 days, and thereafter did not evolve significantly. Chen et al. attributed the initial produced VFA due to the hydrolysis of the inoculum [34]. The main VFA produced during this period was acetic acid. Similarly, an accumulation of VFA, including acetic acid, in a range of 0.03–1.3 g/L was observed during the acclimation of biological methanation communities, as previously reported [19,29,37]. Such accumulation was attributed to the homoacetogenic microbial activity. In these studies, a decrease of the acetoclastic methanogens that convert acetate to CH_4_ was observed, which could explain the acetate accumulation through a preferred pathway of homoacetogenesis and hydrogenotrophic methanogenesis. As an explanation, all reactors started with a H_2_ partial pressure higher than 1 bar could have inhibited acetoclastic methanogens. Indeed, acetoclastic methanogens could be inhibited with only 2.5 mbars of H_2_ [38,39].

### 2.2. Effect of Temperature on the Microbial Community of the Reactors

For all the reactors and whatever the temperature or the inoculum origin, the quantification of the *Archaea* by qPCR showed no difference between the amount of *archaea* in the initial inocula and at the end of the experiments. Illustratively, the *archaea* quantity remained constant at 6.01 × 10^8^ ± 2.37 × 10^7^ and 1.80 × 10^9^ ± 7.21 × 10^8^ 16S rRNA *archaea*/mL for the mesophilic inoculum operated at 35 °C (data in Supplementary Materials). Nonetheless, the 16S rRNA gene sequencing results showed substantial shifts in archaeal composition between the initial inocula and the final microbial communities at the end of the experiments. 

**Figure 4:**
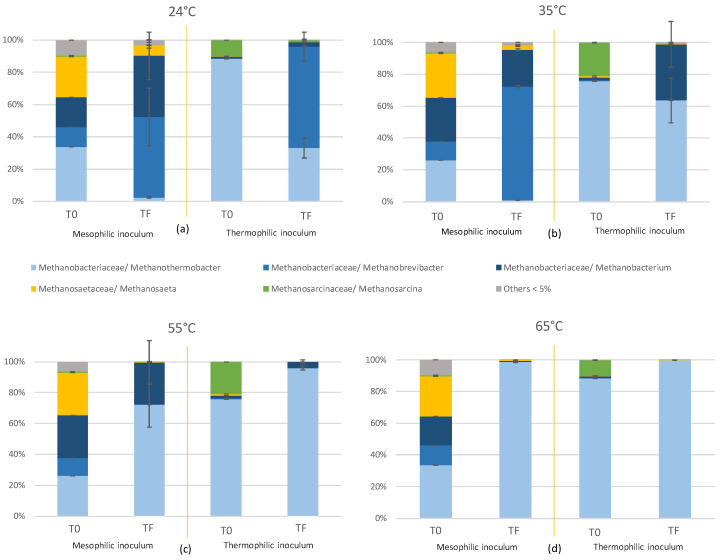
Distribution of the different species of *Methanobacteriacaea*, *Methanosaetaceae* and *Methanosarcinaceae* families present in relative abundance according to temperature. Top left (**a**) for 24 °C, top right (**b**) for 35 °C, bottom left (**c**) for 55 °C and bottom right (**d**) for 65 °C. T0 means the initial composition of the inoculum and TF the composition at the end of the experiment (cycle 14). The two bars on the left of the vertical yellow line are the same represent results for the original inoculum from a 35 °C reactor and those on the right for the original inoculum from a 55 °C reactor.

The microorganisms distribution in the inoculum is shown in Figure 4, with the relative abundance for the species found at more than 5%. First, the composition in *Archaea* in both inocula (day 0) was mainly dominated by hydrogenotrophic methanogens (*Methanobacteriales* order: between 66% and 80%) followed by acetoclastic methanogens (*Methanosarcinales* order: between 10% and 28%). Among the microorganisms involved in biological methanation, some are related to strictly hydrogenotrophs such as members of the genera *Methanothermobacter*, *Methanobrevibacter* and *Methanobacterium* which belong to the *Methanobacteriales* order [26]. In contrast, some other *archaea* were affiliated to strict acetotrophic methanogens such as *Methanosaeta* sp. (*Methanosarcinales* order) [40]. Nontheless, some other genera of *Methanosarcinales* such as *Methanosarcina* sp. can convert different substrates [25]. Therefore, the *Methanobacteriaceae* family from the *Methanobacteriales* order corresponds here to microorganisms that only used hydrogen to produce methane, while *Methanosaetaceae* can use acetate and *Methanosarcinaceae* can use acetate or H_2_ (both in *Methanosarcinales* order) [40]. The comparison between the initial point and the final point of Figure 4a–d) showed an increase in the relative abundance of *Methanobacteriales* over the experimental time and a decrease of *Methanosarcinales*. Such increase in the abundance of hydrogenotrophic methanogens indicated a shift of the principal metabolic pathway towards hydrogenotrophic methanogenesis, as already reported in other biological methanation works [8,18,19,29,33,35,36,41]. This decreased in the number of different orders was also observed by Braga Nan et al. and this was not correlated with the inoculum composition [8].

Guneratnam et al. observed a lack of acetotrophic methanogens and assumed that carbon limited thermophilic conditions could inhibit acetotrophs [29]. As mentioned previously, the high partial pressure of H_2_ and the low acetate concentration in the reactors could also have negatively affected acetotrophs [18,42,43]. 

The abundance in hydrogenotrophic methanogenic *archaea* was higher with the increasing temperature, *Methanosarcinales* abundance decreased at less than 1% at 55 and 65 °C. Dong et al. observed that thermophilic and extreme-thermophilic conditions caused a shift in methanogenesis pathway with the hydrogenotrophic methanogenesis that was privileged over acetoclastic methanogenesis and the archaeal community was dominated by hydrogenotrophic methanogens [32]. *Methanosarcinales* decreased from 81.2% to 50.5% at 55 °C and were not detected at 65 and 70 °C. At 24 °C, the H_2_ solubility was higher and the selection of hydrogenotrophic methanogens, *Methanosaeta* sp. and *Methanosarcina* sp., with a lower H_2_ affinity seems to be favoured [8]. Moreover, the high H_2_ solubility has led to a higher H_2_ partial pressure which favours homoacetogens and inhibit synthrohic bacteria. The VFA production by homoacetogens was favoured but the degradation of VFA by syntrophic bacteria was negatively affected.

By focusing on the genus distribution within the *Methanobacteriaceae* family, *Methanothermobacter* sp., *Methanobrevibacter* sp. and *Methanobacterium* sp. corresponded to the main species (Figure 4). These three *archaea* are hydrogenotrophic methanogens. The number of dominant microorganisms decreased with the increasing temperature. At 24 °C and 35 °C, *Methanobrevibacter* sp. and *Methanobacterium* sp. developed in higher proportion than *Methanothermobacter* sp. At 35 °C, between 24 and 35% of the *Methanobacteriacaea* were represented by *Methanobacterium* sp., and at 55 °C between 5 and 25%. At 37 °C, Tang et al. observed as well between 32 and 45% of *Methanobacterium* sp. [33]. The abundance of *Methanobrevibacter* sp. ranged between 1% and 71% at 35 °C and between 0.5 and 1% at 55 °C. In contrast, *Methanothermobacter* sp. dominated the community at 55 and 65 °C, with an abundance ranging between 72.1% and 99.8%. As reported elsewhere, *Methanothermobacter* sp. dominated biological methanation at a temperature above 50 °C [29,30,36,44]. Recently, Dong et al. and Chen et al. observed a dominance of *Methanothermobacter* sp., with 82.4% at 35 °C within *Methanobacteriales* [32], and of *Methanobacterium* sp. under thermophilic and extreme-thermophilic conditions [32,34].

With regard to the different origins of the inoculum, the initial mesophilic inoculum had a higher diversity of *archaea* than the thermophilic inoculum. That probably contributed to the fast adaptation of the mesophilic inoculum at high temperatures. While *Methanobrevibacter* sp. dominated at 24 °C, at 35 °C, *Methanobrevibacter* sp. dominated and *Methanobacterium* sp. abundance decreased. In comparison, with the thermophilic inoculum, *Methanothermobacter* sp. dominated with an increase of *Methanobacterium* sp. at 35 °C. At 55 °C, with the mesophilic inoculum, *Methanobacterium* sp. was still present although with the thermophilic inoculum, its abundance was low. In counterpart, *Methanobrevibacter* sp. was likely more sensitive to high temperature than *Methanobacterium* sp. In fact, the optimum growth temperature of *Methanobacterium* sp. is 65–70 °C [34]. 

With regard to the repartition of the acetotrophic methanogens, the family *Methanosarcinaceae* was the most abundant acetotrophs in the initial thermophilic inoculum and *Methanosaetaceae* was the most abundant in the initial mesophilic inoculum. After hydrogen injection, for the mesophilic inoculum *Methanosaeta* sp. (*Methanosaetaceae*) remained the unique acetotrophic methanogen. For the thermophilic inoculum, changes were dependent of the temperature. At 24 °C, *Methanosarcina* sp. (*Methanosarcinaceae*) remained the most abundant. At 35, 55 and 65 °C, the proportion between *Methanosaeta* sp. and *Methanosarcina* sp. became negligible.

Zabranska and Pokorna found that *Methanosarcina* sp. decreased with lower concentration of VFA and *Methanosaeta* sp was not observed under thermophilic conditions [25]. Interestingly, the decrease in *Methanosaeta* sp. abundance at low temperature was also probably due to the high hydrogen pressure [38,39]. In contrary, Braga Nan et al. suggested that the presence of *Methanosaeta* sp. and *Methanosarcina* sp. contributed to avoid acetate accumulation and favour methane production. In view of the analyses below, injection of H_2_ leads to a strong selection of hydrogenotrophic methanogens and a sharp decrease in microbial diversity. Hydrogenotrophic methanogenesis was the main metabolism in biological methanation and was mostly achieved by the genus *Methanothermobacter* sp. at high temperatures. Likely due to a higher initial microbial diversity, the mesophilic inoculum adapted more quickly to a change in a wide range of temperatures. 

A correlation between maximal CH_4_ production rate and the increase in *Methanothermobacter* sp. abundances was thus clearly shown. The high abundance in *Methanothermobacter* sp was highly favourable to the biological methanation process with high methane production rate from H_2_/CO_2_. In particular, high *Methanothermobacter* sp abundances were found at thermophilic temperatures. Interestingly, the instability of the system increased also with the increasing temperature. Therefore, the presence of only *Methanothermobacter* sp. at 65 °C probably caused the drop in CH_4_ production after the cycle 10 and therefore the instability of the system.

## 3. Materials and Methods 

### 3.1. Inocula and Nutrient Medium

The microbial inocula were sampled from two different industrial liquid anaerobic digesters operated at mesophilic (35 °C) and thermophilic (55 °C) temperatures. The two full-scale plant digesters were fed with the same sewage sludge from a wastewater treatment plant (France). These inocula were named “mesophilic inoculum” and “thermophilic inoculum”.

The volatile suspended solid (VSS) of each inoculum were measured and the reactors were prepared by diluting these inocula with liquid medium to obtain a concentration of 5 gVSS/L.

A sodium phosphate (0.5 M) solution was used to buffer the medium to a pH of 7.5. A mineral medium was used to provide macro-elements and was composed by: NH_4_Cl 859 mg/L, KH_2_PO_4_ 323 mg/L, MgCl_2_ hexahydrate 194 g/L, CaCl_2_ dihydrate 97 mg/L. The reacting medium was supplemented with an oligo-element solution composed as follows: FeCl_2_ tetrahydrate 20 mg/L, CoCl_2_ hexahydrate 5 mg/L, MnCl_2_ hexahydrate 1 mg/L, NiCl_2_ hexahydrate 1 mg/L, ZnCl_2_ 0.5 mg/L, H_3_BO_3_ 0.5 mg/L, Na_2_SeO_3_ 0.5 mg/L, CuCl_2_ dihydrate 0.4 mg/L, Na_2_MoO_4_ dihydrate 0.1 mg/L.

### 3.2. Reactor Setup and Operation

Schott flasks (600 mL with a working volume of 200 mL) were sealed with rubber stoppers. They were mixed with a magnetic stirbar at a rotation speed of 210 rpm. 

Four different temperatures were tested: 24, 35, 55 and 65 °C. Experiments were performed in two different runs: run 1 at temperatures of 35 and 55 °C and run 2 at temperatures of 24 and 65 °C. As a consequence, the mesophilic and thermophilic inocula were collected twice: a first time for the run 1 and a second time for the run 2. 

For each operating temperature, the two inocula (i.e., mesophilic inoculum and thermophilic inoculum) were used to seed the reactors. For each condition, a biological methanation reaction was carried out by adding a gas composed of a mixture of H_2_/CO_2_ at a ratio of 4:1 in duplicate. A blank reactor (no feeding) was also carried out in duplicate. The gas injection in the head space was performed by pulses, i.e., in a semi continuous regime. For the first substrate injection the head space of the flasks was flushed with a gas mixture of H_2_/CO_2_, until a pressure of 1.5 bars was reached. For other pulses, as soon as the pressure of the vials dropped below 1 bar the gas mixture was injected to reach 1 bar. All injections were performed at ambient temperature (approximately 20 °C). 

After each gas injection, a cycle of operation started. The duration of the cycle was on average 37 h, until the pressure of the reactors was below 1 bar. During the cycle, the following analyses were performed: biogas pressure and composition, volatile fatty acids (VFAs) composition and microbial community composition. The amounts of VFA are given in COD equivalents, considering the following coefficients for VFA/COD conversion: 1.07 g COD/g acetate, 1.51 g COD/g propionate, 1.81 g COD/g butyrate and isobutyrate, 2.04 g COD/g isovalerate and 2.207 g COD/g caproate. Fourteen cycles were achieved for each run. 

Pressure and composition of the gas in head space were analyzed several times per cycle in order to obtain a rate of consumption of H_2_ and the maximal CH_4_ production rate (mL of CH_4_ per L of reactor per day). Blank reactors were used to evaluate the endogenous CH_4_ produced from the degradation of the residual organic matter contained in the inoculum. Methane production rates of the blank reactors were subtracted from the ones of the biological methanation reactors.

Before each gas pulse, 2 mL of the liquid medium were sampled for further analyses of VFA and microbial community compositions.

### 3.3. Analytical Methods

Biogas composition (H_2_, O_2_, N_2_ and CH_4_) was determined using a Clarus 580 gas chromatograph (Perkin Elmer, Waltham, MA USA) equipped with two capillary columns Rt Q-Bond (30 m × 0.32 mm × 10 µm) and a Molsieve 5A (30 m × 0.32 mm × 30 µm) and thermal conductivity detector (TCD). Argon was used as a carrier gas at a pressure of 3.5 bar. Gas pressure was measured manually with a manometer Keller LEO 2 (KELLER AG, Winterthur, Switzerland) as reported elsewhere [45]. The amount of component i (n_i_, in mole) in the bottles was calculated according to the following equation:

(2) $$ni=\;\frac{P\times xi\times Vh}{8.314\times \left(273.15+T\right)}$$

where *xi* is to the percentage of component *i* in the biogas (given by CPG measure), *P*_total_ the total pressure (Pa), *V_h_* is the headspace volume (m^3^), 8.314 is the universal gas constant, and *T* the temperature (°C). 

Liquid samples were first centrifuged and the liquid fraction was used to analyze the VFA production while the pellet was kept at −20 °C to further molecular analysis. Volatile fatty acids (VFA) composition in the liquid phase was determined using a Clarus 580 gas chromatograph (Perkin Elmer) equipped with an Elite-FFAP cross-bond^®^ carbowax^®^ capillary column (15 m × 0.53 mm) and flame ionization detection (FID). The detector temperature was set at 280 °C, N_2_ was used as gas carrier at 6 mL min^−1^ as reported by Cazier et al. [39]. 

### 3.4. Statistical Analysis

Descriptive statistics were carried out to calculate mean values and standard deviations of the maximal CH_4_ production rate (mL of CH_4_ per liter of reactor per day). The cycles 10 and 8 for the run 1 (35 and 55 °C) and 2 (24 and 65 °C), respectively, were not considered for the statistical analyses as a change of septum was performed at the beginning of these cycles. In fact, the gas composition of the headspace was modified and it strongly affected the CH_4_ production rate (Figure 1). In addition, the first three cycles were also excluded, since the inocula were rich in residual organic matters at the beginning of the experiment. A comparison of the maximal CH_4_ production rates between the different operating conditions (i.e., temperature and inoculum) was achieved. The normality of the data and the homogeneity of variance were analyzed in order to determine the statistical test required. The normality of the data was confirmed by a Shapiro test. For each temperature data set two inocula so two groups were compared, F-test is performed to check equality of variances and then a student t-test was used to compare the effect of the two inocula for each temperature. A correction was performed on the *p* values with false discovery rate since the data were separated by temperature. Because there were more than two groups to compare a different test was done to compare the 4 temperatures. A Levene test was performed to check the homogeneity of the variances first, they were not homogeneous therefore a Kruskal Wallis test was done to know if, at less, one of the temperature was different from the others. A Kruskal Wallis test was performed on each temperature data set. Then the Dunn test compared temperatures two by two. The correction of Benjamini-Hochberg was added to the Dunn test to correct the *p* values since the data were compared one temperature to another. 

### 3.5. DNA Extraction and Sequencing 

To analyze the microbial community composition and dynamics, Illumina Miseq sequencing and real time PCR methods were performed. Two samples were analyzed for all reactors: the initial inoculum and the last-day-of-operation sample. DNA extraction was made with FastDNA™ SPIN kit in accordance with the manufacturer’s instructions (MP Biomedicals LCC, Santa Ana, CA, USA). Infinite 200 PRO NanoQuant (Tecan Group Ltd., Männedorf, Switzerland) was used to quantify the extracted DNA concentration.

For the identification of the *Archaea* community, amplicons from the V4 to V5 regions of 16S rRNA genes were amplified with degenerated primers designed by our laboratory: 5′-CAGMGCCGCGGKAA-3′ (F504–F519) and 5′-CCCGCCWATTCCTTTAAGT-3′ (R910–R928). The PCR mixtures (60 μL) contained 0.05 u/uL of MTP™ Taq DNA Polymerase (Sigma-Aldrich, Inc., Merck, Darmstadt, Germany) with its corresponding buffer, 0.5 mM of each primer (forward and reverse), 0.2 mM of each dNTP and between 0.04 to 0.2 ng/uL of genomic DNA. Reactions were carried out in a Mastercycler^®^ thermal cycler (Eppendorf, Hamburg, Germany) as follows: Taq polymerase activation at 95 °C for 2 min, followed by 35 cycles of denaturation at 95 °C for 1 min, annealing at 59 °C for 1 min, and elongation at 72 °C for 1 min and a final extension at 72 °C for 10 min. PCR products were confirmed by 2100 Bioanalyzer (Agilent, Santa Clara, CA, USA). The community composition was sequenced in Illumina Miseq sequencer at the GenoToul platform, Toulouse, France (www.genotoul.fr). Mothur version 1.39.5 was used to reads cleaning, assembly and quality checking. For alignment and as taxonomic outline SILVA release 128 was used.

### 3.6. Quantitative PCR

The amplification qPCR programs were performed in BioRad CFX96 Real-Time Systems C1000 Touch Thermal Cycler (Bio-Rad Laboratories, Foster city, CA, USA). For total *Archaea* qPCR analysis, primers 787F and 1059R were used [46]. qPCR reactions were carried using the following mix: 12.5 μL SsoAdvanced™ Universal Probes Supermix (Bio-Rad Laboratories), 200 nM of the 787F primer and 1059R primer, 50 nM TaqMan probe, 5 μL of a DNA sample dilution and water were added to obtain a final volume of 12.5 μL for all analyses. A first incubation of 2 min at 95 °C followed by 40 cycles of denaturation (95 °C, 15 s) and hybridation-elongation (60 °C, 1 s) was performed. Two different dilutions of each DNA samples were analyzed in triplicates. 

## 4. Conclusions

The aim of this study was to evaluate the effect of the temperature on the ex-situ biological methanation, more specifically on microbial activities and reactor performances. The inoculum with mesophilic origins acclimated rapidly to high temperatures and showed similar performances to the inoculum of thermophilic origin at the same operating temperatures. For both inocula, the maximal methane production rates were higher at thermophilic temperatures with a concomitant higher *Methanothermobacter* sp. abundance, even if the instability of the system increased. Such instability was probably due to the unique dominance of *Methanothermobacter* sp. at high temperatures. At 35 °C, the maximal methane production rate was higher with mesophilic inoculum and the inoculum origin had no influence at 55 °C. Considering both the methane production rate and the system stability, a temperature range from 35 °C to 55 °C is recommended to achieve ex-situ biological methanation. 

## Figures and Tables

**Table 1:** Reactor performances listed by temperatures then by type of inoculum, which includes the cycles selected for the statistical analysis. Line 3 and 4 contain the initial and final pH values. Lines 5 and 6 represent the average CH_4_ production rate and H_2_ consumption rate calculated with the maximal CH_4_ production rate of each cycle and the maimal H_2_ consumption rate of each cycle. Lines 7 and 8 correspond to the average of final total VFA concentration and final acetate concentration. Line 9 is the ratio of the quantity of VFA (in g CODeq) cumulated in regards to the quantity of CH_4_ and VFA (in g CODeq) cumulated.

Temperature	24 °C		35 °C		55 °C		65 °C	
Inoculum Origin	Mesophilic	Thermophilic	Mesophilic	Thermophilic	Mesophilic	Thermophilic	Mesophilic	Thermophilic
Initial pH	7.75	7.57	7.48	7.40	7.45	7.42	7.73	7.57
Final pH	7.37	7.27	7.78	7.95	8.01	7.90	7.86	7.68
CH_4_ production rate (mL/L.day)	156 ± 41	112 ± 37	253 ± 51	213 ± 48	290 ± 55	283 ± 75	309 ± 109	304 ± 82
H_2_ consumption rate (mL/L.day)	773 ± 119	643 ± 135	826 ± 109	734 ± 118	994 ± 167	935 ± 178	900 ± 368	856 ± 310
Final VFA concentration (g/L)	1.130 ± 0.260	0.790 ± 0.590	0.965 ± 0.042	1.220 ± 0.130	1.030 ± 0.001	1.640 ± 0.220	0.680 ± 0.040	0.340 ± 0.006
Final acetate concentration (g/L)	0.911 ± 0.185	0.669 ± 0.570	0.639 ± 0.054	0.848 ± 0.102	0.595 ± 0.010	1.140 ± 0.151	0.361 ± 0.015	0.164 ± 0.001
Cumulated VFA / (Cumulated VFA + Cumulated CH_4_) (%)	15 ± 2	14 ± 1	−3 ± 2	3 ± 1	0 ± 2	2 ± 1	2 ± 3	1 ± 0

## Supplementary Materials

The following are available online, Table S1: Quantification of the *Archaea* by qPCR with 16S rRNA. Average quantity of 16S rRNA per mL of sample and standard deviation are given for duplicates at the first cycle 0 and at the final cycle of reactors with H_2_ addition. Mesophilic-24-P0 corresponds to the initial microbial quantity for the mesophilic inoculum collected for the run 2. Mesophilic-35-P0 corresponds to the initial microbial quantity for the mesophilic inoculum collected for the run 1. Thermophilic-24-P0 corresponds to the initial microbial quantity for the mesophilic inoculum collected for the run 2. Thermophilic-35-P0 corresponds to the initial microbial quantity for the mesophilic inoculum collected for the run 1, Figure S1: Methane production correlated with hydrogen consumption, Table S2: Phylogenetic overview of abundant taxonomic guilds. The 10 most abundant *archaea*, with percentage superior at 1, at genus level. Average abundance and standard deviation are given for duplicates at the first cycle 0 and at the final cycle of reactors with H_2_ addition. Mesophilic-24-P0 corresponds to the initial microbial community of the mesophilic inoculum collected for the run 2. Mesophilic-35-P0 corresponds to the initial microbial community of the mesophilic inoculum collected for the run 1. Thermophilic-24-P0 corresponds to the initial microbial community of the mesophilic inoculum collected for the run 2. Thermophilic-35-P0 corresponds to the initial microbial community of the mesophilic inoculum collected for the run 1.
