# Supplementary material for: Temperature and Inoculum Origin Influence the Performance of Ex-Situ Biological Hydrogen Methanation
Source: Molecules. 2020 Dec 1;25(23):5665. doi: 10.3390/molecules25235665 (PMC7730501; doi:10.3390/molecules25235665)
Supplement: Supplementary file 1 [file molecules-25-05665-s001.pdf]

Supplementary Materials

Temperature and Inoculum Origin Influence the Performance of *ex-situ* Biological Hydrogen Methanation

Noémie Figeac [1,2], Eric Trably [1], Nicolas Bernet [1], Jean-Philippe Delgenès [1] and Renaud Escudié [1,*]

[1] INRAE, University of Montpellier, LBE, 102 avenue des Etangs, 11100 Narbonne, France; noemie.figeac@inrae.fr (N.F.) ; eric.trably@inrae.fr (E.T.); nicolas.bernet@inrae.fr (N.B.); jean-philippe.delgenes@inrae.fr (J.-P.D.)

[2] French Environment and Energy Management Agency, 20 avenue du Grésillé- BP 90406 49004 Angers Cedex 01, France;

* Correspondence: renaud.escudie@inrae.fr; Tel.: +33-4684-251-73

Table S1 : Quantification of the Archaea by qPCR with 16S rRNA. Average quantity of 16S rRNA per mL of sample and standard deviation are given for duplicates at the first cycle 0 and at the final cycle of reactors with H2 addition. Mesophilic-24-P0 corresponds to the initial microbial quantity for the mesophilic inoculum collected for the run 2. Mesophilic-35-P0 corresponds to the initial microbial quantity for the mesophilic inoculum collected for the run 1. Thermophilic-24-P0 corresponds to the initial microbial quantity for the mesophilic inoculum collected for the run 2. Thermophilic-35-P0 corresponds to the initial microbial quantity for the mesophilic inoculum collected for the run 1.

	Mesophilic-24-P0	Mesophilic-24-PF	Thermophilic-24-P0	Thermophilic-24-PF	Mesophilic-35-P0	Mesophilic-35-PF	Thermophilic-35-P0	Thermophilic-35-PF	Mesophilic-55-P0	Mesophilic-55-PF	Thermophilic-55-P0	Thermophilic-55-PF	Mesophilic-65-P0	Mesophilic-65-PF	Thermophilic-65-P0	Thermophilic-65-PF
16S rRNA archaea / ml sample	$5.24 \times 10^{08} \pm 4.48 \times 10^{07}$	$9.14 \times 10^{08} \pm 5.45 \times 10^{07}$	$4.76 \times 10^{08} \pm 2.67 \times 10^{07}$	$9.34 \times 10^{08} \pm 4.60 \times 10^{07}$	$6.01 \times 10^{08} \pm 2.37 \times 10^{07}$	$1.80 \times 10^{09} \pm 7.21 \times 10^{07}$	$9.47 \times 10^{08} \pm 9.68 \times 10^{07}$	$7.93 \times 10^{08} \pm 7.25 \times 10^{07}$	$6.01 \times 10^{08} \pm 2.37 \times 10^{07}$	$9.81 \times 10^{08} \pm 4.49 \times 10^{07}$	$9.47 \times 10^{08} \pm 9.68 \times 10^{07}$	$9.50 \times 10^{08} \pm 8.63 \times 10^{07}$	$5.24 \times 10^{08} \pm 4.48 \times 10^{07}$	$5.83 \times 10^{08} \pm 2.16 \times 10^{07}$	$4.76 \times 10^{08} \pm 2.67 \times 10^{07}$	$1.13 \times 10^{09} \pm 1.03 \times 10^{08}$

T

Table S2: Phylogenetic overview of abundant taxonomic guilds. The 10 most abundant archaea, with percentage superior at 1, at genus level. Average abundance and standard deviation are given for duplicates at the first cycle 0 and at the final cycle of reactors with H2 addition. Mesophilic-24-P0 corresponds to the initial microbial community of the mesophilic inoculum collected for the run 2. Mesophilic-35-P0 corresponds to the initial microbial community of the mesophilic inoculum collected for the run 1. Thermophilic-24-P0 corresponds to the initial microbial community of the mesophilic inoculum collected for the run 2. Thermophilic-35-P0 corresponds to the initial microbial community of the mesophilic inoculum collected for the run 1.

	Mesophilic-24-P0	Mesophilic-24-PF	Thermophilic-24-P0	Thermophilic-24-PF	Mesophilic-35-P0	Mesophilic-35-PF	Thermophilic-35-P0	Thermophilic-35-PF	Mesophilic-55-P0	Mesophilic-55-PF	Thermophilic-55-P0	Thermophilic-55-PF	Mesophilic-65-P0	Mesophilic-65-PF	Thermophilic-65-P0	Thermophilic-65-PF
Methanothermobacter	9,0% ± 0,0%	1,9% ± 0,5%	88,3% ± 0,0%	33,0%± 6,3%	6,1%± 0,0%	0,7%± 0,0%	75,7%± 0,0%	63,6%± 14,2%	6,1%± 0,0%	72,1%± 14,4%	75,7%± 0,0%	95,9%± 1,3%	9,0%± 0,0%	98,6%± 0,1%	88,3%± 0,0%	99,8%± 0,0%
WCHA1-57_ge	24,6% ± 0,0%	0,5%± 0,0%	0,1%± 0,0%	0,0%± 0,0%	19,9%± 0,0%	0,2%± 0,1%	0,1%± 0,0%	0,0%± 0,0%	19,9%± 0,0%	0,0%± 0,0%	0,1%± 0,0%	0,0%± 0,0%	24,6%± 0,0%	0,1%± 0,0%	0,1%± 0,0%	0,0%± 0,0%
Methanobrevibacter	12,4%± 0,0%	49,8%± 17,9%	0,4%± 0,0%	63,0%± 8,9%	11,7%± 0,0%	71,4%± 0,7%	0,7%± 0,0%	0,3%± 0,2%	11,7%± 0,0%	0,1%± 0,0%	0,7%± 0,0%	0,0%± 0,0%	12,4%± 0,0%	0,1%± 0,0%	0,4%± 0,0%	0,0%± 0,0%
Methanobacterium	18,3%± 0,0%	37,9%± 14,7%	0,7%± 0,0%	2,5%± 1,8%	27,4%± 0,0%	23,2%± 0,7%	1,7%± 0,0%	34,9%± 14,5%	27,4%± 0,0%	27,2%± 14,3%	1,7%± 0,0%	3,9%± 1,3%	18,3%± 0,0%	0,7%± 0,1%	0,7%± 0,0%	0,0%± 0,0%
Methanosaeta	25,4%± 0,0%	6,5%± 1,8%	0,5%± 0,0%	0,2%± 0,0%	27,9%± 0,0%	2,6%± 0,6%	0,9%± 0,0%	0,3%± 0,1%	27,9%± 0,0%	0,5%± 0,1%	0,9%± 0,0%	0,1%± 0,0%	25,4%± 0,0%	0,5%± 0,0%	0,5%± 0,0%	0,1%± 0,0%
Methanosarcina	0,4%± 0,0%	0,1%± 0,0%	9,9%± 0,0%	1,1%± 0,7%	0,6%± 0,0%	0,0%± 0,0%	20,9%± 0,0%	0,0%± 0,0%	0,6%± 0,0%	0,0%± 0,0%	20,9%± 0,0%	0,0%± 0,0%	0,4%± 0,0%	0,0%± 0,0%	9,9%± 0,0%	0,1%± 0,0%
Methanoculleus	2,1%± 0,0%	0,6%± 0,3%	0,0%± 0,0%	0,0%± 0,0%	0,2%± 0,0%	1,1%± 0,5%	0,0%± 0,0%	0,9%± 0,0%	0,2%± 0,0%	0,0%± 0,0%	0,0%± 0,0%	0,0%± 0,0%	2,1%± 0,0%	0,0%± 0,0%	0,0%± 0,0%	0,0%± 0,0%
Methanospirillum	4,1%± 0,0%	1,9%± 0,4%	0,1%± 0,0%	0,0%± 0,0%	3,5%± 0,0%	0,3%± 0,1%	0,0%± 0,0%	0,0%± 0,0%	3,5%± 0,0%	0,0%± 0,0%	0,0%± 0,0%	0,0%± 0,0%	4,1%± 0,0%	0,1%± 0,0%	0,1%± 0,0%	0,0%± 0,0%
Methanosphaera	1,0%± 0,0%	0,1%± 0,0%	0,0%± 0,0%	0,0%± 0,0%	1,8%± 0,0%	0,0%± 0,0%	0,0%± 0,0%	0,0%± 0,0%	1,8%± 0,0%	0,0%± 0,0%	0,0%± 0,0%	0,0%± 0,0%	1,0%± 0,0%	0,0%± 0,0%	0,0%± 0,0%	0,0%± 0,0%
Candidatus_Methanoplasma	1,3%± 0,0%	0,2%± 0,0%	0,0%± 0,0%	0,0%± 0,0%	0,6%± 0,0%	0,0%± 0,0%	0,0%± 0,0%	0,0%± 0,0%	0,6%± 0,0%	0,0%± 0,0%	0,0%± 0,0%	0,0%± 0,0%	1,3%± 0,0%	0,0%± 0,0%	0,0%± 0,0%	0,0%± 0,0%
Others < 1%	1,3%± 0,0%	0,6%± 0,0%	0,0%± 0,0%	0,2%± 0,0%	0,5%± 0,0%	0,4%± 0,0%	0,0%± 0,0%	0,1%± 0,1%	0,5%± 0,0%	0,0%± 0,0%	0,0%± 0,0%	0,0%± 0,0%	1,3%± 0,0%	0,0%± 0,0%	0,0%± 0,0%	0,0%± 0,0%
